# Analysis and Optimization of Trapezoidal Grooved Microchannel Heat Sink Using Nanofluids in a Micro Solar Cell

**DOI:** 10.3390/e20010009

**Published:** 2017-12-25

**Authors:** Ruijin Wang, Wen Wang, Jiawei Wang, Zefei Zhu

**Affiliations:** School of Mechanical Engineering, Hangzhou Dianzi University, Hangzhou 310018, China

**Keywords:** micro solar cell, nanofluid, microchannel heat sink, heat transfer enhancement, numerical simulation

## Abstract

It is necessary to control the temperature of solar cells for enhancing efficiency with increasing concentrations of multiple photovoltaic systems. A heterogeneous two-phase model was established after considering the interacting between temperature, viscosity, the flow of nanofluid, and the motion of nanoparticles in the nanofluid, in order to study the microchannel heat sink (MCHS) using Al_2_O_3_-water nanofluid as coolant in the photovoltaic system. Numerical simulations were carried out to investigate the thermal performance of MCHS with a series of trapezoidal grooves. The numerical results showed us that, (1) better thermal performance of MCSH using nanofluid can be achieved from a heterogeneous two-phase model than that from single-phase model; (2) The effects of flow field, volume fraction, nanoparticle size on the heat transfer enhancement in MCHS were interpreted by a non-dimensional parameter N_BT_ (i.e., ratio of Brownian diffusion and thermophoretic diffusion). In addition, the geometrical parameters of MCHS and the physical parameters of the nanofluid were optimized. This can provide a sound foundation for the design of MCHS.

## 1. Introduction

With the rapid increase of concentrated multiples in the photovoltaic system, to improve the efficiency of solar cell is an issue of concern. An effective approach for decreasing the surface temperature of photovoltaic module is to introduce a specialized cooling system [1,2,3,4]. Hence, a variety of approaches are utilized to cool the solar cell, the most frequently used are jet-impact cooling and microchannel heat sink (MCHS) [5,6,7,8]. Great interests were aroused by the excellent heat transfer performance of MCHS [9,10,11,12,13,14]. The use of nanofluid as a coolant can further promote the heat transfer performance of MCHS [15,16,17,18]. Most research work has focused on the MCHS layout, geometrical parameters of microchannel, and so on. Osman et al. [19] investigated the influence of the layout and arrangement of microchannels on thermal performance. P. Gunnasegaran [20] studied the thermal performance of various MCHS with various cross-section (rectangle, trapezoidal, circle, and ellipse). In order to improve the heat transfer performance, various grooved or ribbed microchannels are often applied. However, the grooved microchannels were used more frequently than ribbed one because of the smaller flow resistance in such a channel. Hamdi et al. [21] investigated the heat transfer performance of MCHS with V-grooves, rectangle grooves and trapezoidal grooves on the side walls by 3-D numerical simulation. However, they didn’t reveal the mechanism of heat transfer enhancement on the basis of the distribution of the channel flow, especially, of the distribution of boundary layer. The geometrical parameters of the grooves were optimized furthermore. Parameterized investigation on the heat transfer enhancement of the MCHS with slant fins were carried out by Lee [22].

It is known to us that the traditional coolant used in heat transfer is water. However, to meet the requirements of increasing the enlargement of heat flux is hard work for such a coolant. It is an effective way for us to improve the heat transfer performance by adding nanoparticles into the working medium to prepare so-called “nanofluids”. The main reasons for heat transfer enhancement by the suspended nanoparticles in liquid was proved by Xuan [23] to have a greater heat transfer surface between liquid and nanoparticles, increased thermal conductivity, and more collisions between nanoparticles. Unfortunately, there is no unified understanding of the process so far. The likely reasons for heat transfer enhancement could be the Brownian motion of nanoparticles which can thin the heat boundary layer, induce microconvection, or increase the diffusion of nanoparticles. In addition, all of the factors including the interfacial layer around the nanoparticles, aggregation of nanoparticles, and thermophoresis of nanoparticles, and so on, could be regarded as possible mechanisms for improving the thermal conductivity of nanofluids [24,25,26,27,28]. A single-phase model was normally used to numerically simulate the heat and mass transfer in a microchannel in the early phase. A two-phase model, including an Eulerian-Eulerian and an Eulerian-Lagrangian model proved to be more exact [29,30]. Buongiorno [31] established two-components four-equations model based on the dimensional analysis of seven possible micro-mechanisms, and eliminated the underestimation of thermal conductivity of nanofluid in single-phase model or discrete particle model (DPM). Brownian motion and thermophoresis are regarded as the most important factors for heat transfer enhancement of nanofluids. Hereafter, quite a few researchers studied the heat transfer enhancement of nanofluid using Buongiorno’s model, such as Alvarino [32] and Ryzhkov [33]. Nevertheless, the interactions among particle concentration, temperature and flow of nanofluid are not within the consideration in most numerical results, although it is known to us that the viscosity and thermal conductivity of nanofluids will be influenced by the particle distribution induced by the variation of the temperature and the flow of nanofluids. 

It is necessary for us to discover the microscale mechanism of heat transfer enhancement in a MCHS, so as to improve the thermal performance of MCHS used in a micro solar cell. We used numerical investigations for flow and heat transfer in MCHS with periodically arranged trapezoidal grooves on the side wall, on the basis of the establishment of a heterogeneous two-phase model after the consideration of the interaction between the temperature, the viscosity, the flow and the motion of nanoparticles in Al_2_O_3_/water nanofluids. 

## 2. Computational Procedure

### 2.1. Geometrical Model

**Figure 1:**
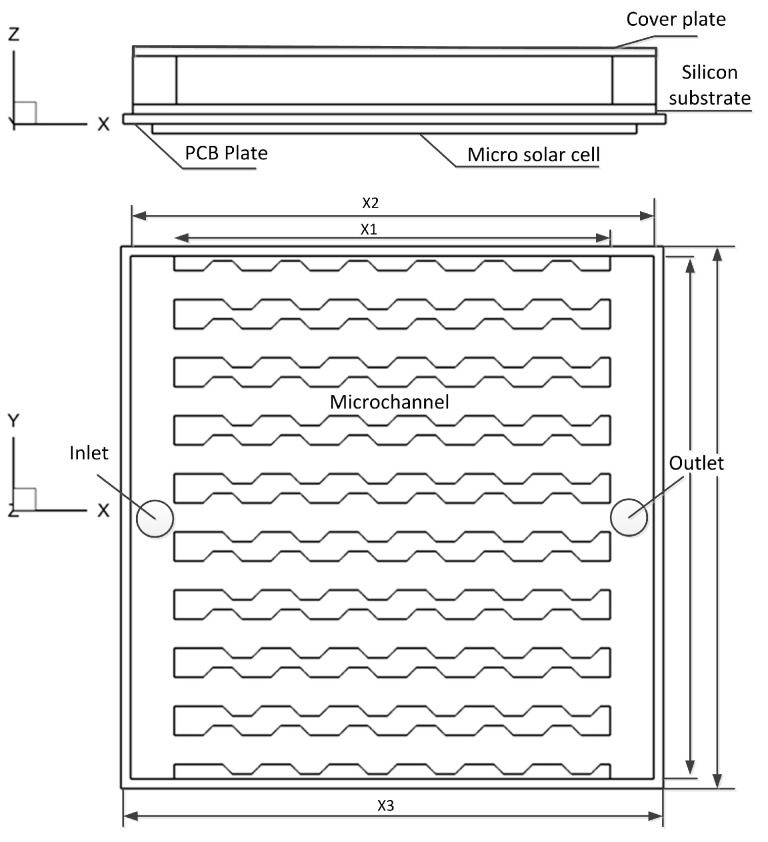
Schematic of microchannel heat sink.

For the micro solar cell composing of photovoltaic modules, silicon substrate and microchannel heat sink (shown in Figure 1), a MCHS model is established. The materials parameters determined according to the technical requirements, and reference [34], are listed in Table 1. The geometrical parameters are as follows: the thickness of the PCB plate and silicon substrate are both 0.1 mm, the overall dimension of MCHS X2 × W2 = 5.4 × 5.4 mm, the height of the microchannel is 0.6 mm. Also, the dimension of the cooling component with nine microchannels (width = 0.3 mm) is X1 × W2 = 4.5 × 5.4 mm, and the channel interval is 0.3 mm. One inlet and one outlet with a diameter of *φ* 0.5 mm are bored in the MCHS.

**Figure 2:**
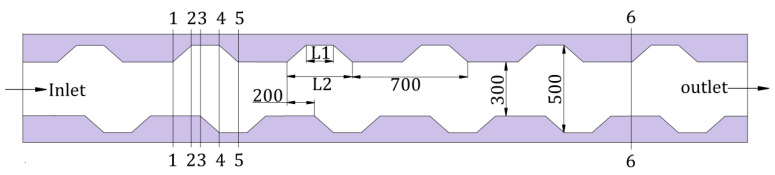
Microchannel with staggered arrangement trapezoidal grooves (nm).

A rectangle microchannel with periodically arranged trapezoidal grooves on the side walls was numerical analyzed, because this MCHS has perfect thermal performance in accordance to the investigation of reference [21]. On the basis of the analysis in [34], the structure dimensions of trapezoidal groove in MCHS were determined as follow: the length of up and bottom side were L1, L2, respectively, the fixed gap of trapezoidal groove was 0.3 mm (i.e., the pitch of groove was 0.7 mm). The staggered arrangement trapezoidal grooves are shown in Figure 2.

### 2.2. Numerical Model

#### 2.2.1. Control Equations

It is known from Qu [35] that, the no-slip Navier-Stokes equation is also valid when the hydraulic diameter being less than 100 μm, and Reynolds number less than 1700. Hence, the control equations of the nanofluid flow in a microchannel, including the mass conservation equation, momentum conservation equation, and energy conservation equation. 

For a steady flow, the mass conservation equation can be written as:

(1) $$\nabla \cdot \vec{V}=0$$

where, $\vec{V}$ is velocity of nanofluid. The momentum conservation equation neglecting gravity reads as:

(2) $$\rho_{nf}[\frac{\partial \vec{V}}{\partial t}+(\vec{V}\cdot \nabla )\vec{V}]=-\nabla p+\mu_{nf}\nabla^{2}\vec{V}$$

where, $\rho_{nf}$ is density of nanofluid, p is pressure, $\mu_{nf}$ is the viscosity of nanofluid. The energy conservation equation considering the influences of Brownian motion and thermophoresis of nanoparticles can be written as [31]:

(3) $$\rho_{nf}c_{nf}\left[\frac{\partial T}{\partial t}+V\cdot \nabla T\right]=\nabla \cdot k_{nf}\nabla T+\rho_{p}c_{p}\left[D_{B}\nabla \varphi \cdot \nabla T+D_{T}\frac{\nabla T\cdot \nabla T}{T}\right]$$

where, T is temperature,c is specific heat, $k_{nf}$ is thermal conductivity, φ is volume fraction of nanoparticle, $D_{B}$ is Brownian diffusion coefficient, $D_{T}$ is thermophoretic diffusion coefficient. The subscript *nf* in the above equations represent nanofluid, *p* represents nanoparticle, *f* represents base fluid (the same below). It is known that the unsteady term in Equations (2) and (3) both are zero for a steady flow. The Brownian diffusion coefficient is related to the environment temperature, the viscosity of base fluid, particle size, and can be read as:

(4) $$D_{B}=\frac{k_{B}T}{3\pi \mu_{f}d_{p}}$$

where, $k_{B}$ = 1.381 × 10^−23^ is the Boltzmann constant, $d_{p}$ is the diameter of nanoparticle. The thermo-phoretic coefficient is proportional to the volume fraction, moreover, it is related not only to the viscosity and density of base fluid, but also to the thermal conductivity of base fluid and nanoparticle [30]. It can be read as:

(5) $$D_{T}=\beta \frac{\mu_{f}}{\rho_{f}}\varphi =0.26\frac{k_{f}}{2k_{f}+k_{p}}\frac{\mu_{f}}{\rho_{f}}\varphi$$

It is worth noting that, the energy transportations of Brownian motion and thermophoresis in energy conservation equation are related to the gradient of volume fraction. Hence, the species conservation equation should be solved. The convective and diffusion equation of species (nanoparticle) including Brownian motion and thermophoresis can be written as:

(6) $$\frac{\partial \varphi }{\partial t}+\vec{V}\cdot \nabla \varphi =\nabla \cdot \left[D_{B}\nabla \varphi +D_{T}\frac{\nabla T}{T}\right]$$

In similar, unsteady term in Equation (6) is zero for steady flow. Equations (1)–(3) and (6) are two-component four-equation models with consideration of Brownian motion and thermophoresis of the nanoparticles, and this model is a heterogeneous two-phase model.

#### 2.2.2. Physical Properties of Nanofluids

The physical properties of nanofluids, such as density, viscosity, specific heat, thermal conductivity, will vary with the flow of the nanofluid, the temperature of the nanofluid, and the nanoparticle concentration of the nanofluid. Table 2 lists the material properties of nanoparticles (Al_2_O_3_) and base fluid (water). The viscosity varying with the temperature can be written as [36]:

(7) $$\mu_{f}=\left(2.414\times 10^{-5}\right)\times 10^{\left(\frac{247.8}{T-140}\right)}$$

For simplification, the Brinkman model for viscosity of nanofluid reads as [37]:

(8) $$\mu_{nf}=\left(1+2.5\varphi \right)\mu_{f}$$

The density and specific heat capacity of nanofluid can be obtained from mixture law [38]:

(9) $$\rho_{nf}=\varphi \rho_{p}+\left(1-\varphi \right)\rho_{f}$$

(10) $${\left(c\rho \right)}_{nf}=\varphi {\left(c\rho \right)}_{p}+\left(1-\varphi \right){\left(c\rho \right)}_{f}$$

The thermal conductivity model of Chon et al. [39] which agrees very well with the experimental results of ${\mathrm{Al}}_{2}O_{3}$-water can be written as:

(11) knfkf=1+64.7φ0.7460(dfdp)0.3690(kpkf)0.7476Pr0.9955Rep1.2321

where, $d_{f}$ = 0.28 nm is the diameter of water molecule, the Prandtl number Pr=cμ/k, the particle Reynolds number Rep=ρfubdp/μf, Brownian velocity of particle $u_{b}=k_{B}T/3\pi \mu_{f}d_{p}\lambda_{f}$, here the mean free path of water molecule $\lambda_{f}$ is set to be 0.17 nm [38]. 

#### 2.2.3. Performance Evaluation Criterion

A suitable index to measure the heat transfer enhancement which is named the Performance Evaluation Criterion (PEC) is needed for the comprehensive consideration of heat convective and pressure loss along the stream. Hence, PEC can be defined as [40]:

(12) $$PEC=\frac{Nu_{nf}/Nu_{f}}{{\left(\Delta P_{nf}/\Delta P_{f}\right)}^{1/3}}$$

Here, ΔP is pressure loss. Nusselt Number Nu, which represents the ability of heat transfer, can be read as:

(13) $$Nu=\frac{hD}{k}$$

where, D is hydraulic diameter defined as the ratio of section area to wetted perimeter, h is heat transfer coefficient between wall of microchannel and working medium, can be defined as:

(14) $$h=\frac{qA_{b}}{A_{ch}\left(T_{c}-T_{f}\right)}$$

where, $A_{b}$ is the heating area on the bottom of microchannel, $A_{ch}$ is the convective area, q is heat flux density, and $T_{c}=\frac{\int TdA}{\int dA}$,$T_{f}=\frac{\int T\rho_{f}dV}{\int \rho_{f}dV}$.

The flow resistance of nanofluid in a microchannel can be expressed by friction coefficient can be defined as:

(15) $$f=\frac{2\Delta PD}{L\rho_{nf}v_{in}^{2}}$$

where, L is the distance of fluid flow, $v_{in}$ is inlet velocity.

### 2.3. Solving the Numerical Model

Contrary to the MCHS used in the micro solar cell, numerical simulations were carried out to obtain a reasonable arrangement for the microchannel. To reveal the influence mechanism of trapezoidal grooves on the heat transfer enhancement, a study on a single microchannel taken from the center of MCHS was conducted, and the structural parameters of trapezoidal groove were optimized.

The process of numerical solution is as follow: geometric modeling → meshing → setting up the boundary condition → compiling the user defined function (UDF) according to Equations (4), (5), and (7)–(10) → calculating by Fluent 12.1 → data outputting → post-processing by Tecplot 10.0.

“No-slip” boundary is enforced, that is neither velocity-slip nor temperature-jump arises at the solid-fluid interface. “Inlet-velocity” in the direction of +x is set, fluid temperature is 293 K, “outflow” is set at outlet. A constant heat flux 544,200 W/m^2^ from the surface of solar cell is set according to reference [41]. All other surfaces are set to be “adiabatic”, and all surface of contact between different material are set to be “interface”.

In consideration of the interaction among the temperature, the viscosity, the flow, and the motion of nanoparticle in Al_2_O_3_/water nanofluid, the viscosity, density, specific heat capacity, thermal conductivity, Brownian, and thermophoretic diffusion coefficient should be changed. For this purpose, the UDF compiled from Equations (4), (5), and (7)–(15) should be invoked in every iteration.

Verification of grid independence was conducted. The discrepancy of the calculated Nusselt number for grid number of 1,070,000, 2,510,000, 3,280,000, 4,340,000 was 4.43%, 1.36%, 0.68%, respectively, when the Reynolds number being 200. Hence, the calculation accuracy is high enough when grid number was bigger than 3,280,000. 

### 2.4. Model Validity 

**Figure 3:**
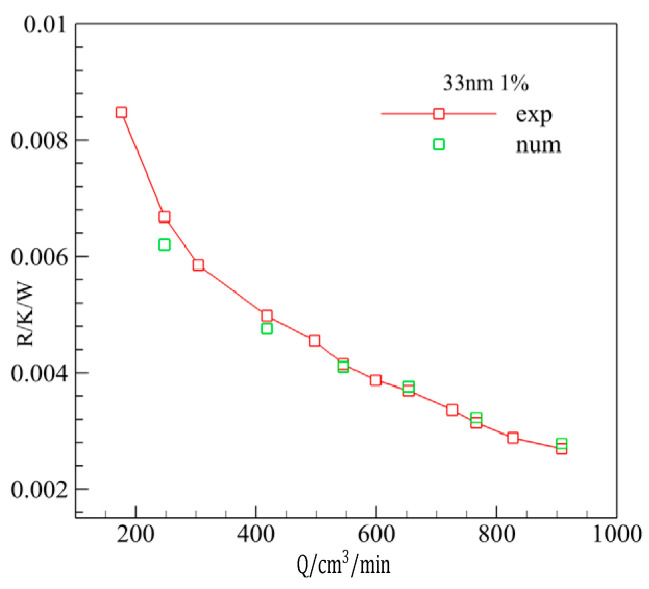
The validity of the numerical model.

The thermal resistances of MCHS using Al_2_O_3_-water were calculated by the above model according to the condition of reference [42]. Good agreement could be found (Figure 3) between the numerical results and experimental results of Ref. [42], especially when the flow rate was larger than 400 cm^3^/min. The biggest deviation value of thermal resistance being 6.43% verified the validity of the present numerical model and the simulation process.

## 3. Results and Discussion

### 3.1. Effect of the Layout

**Figure 4:**
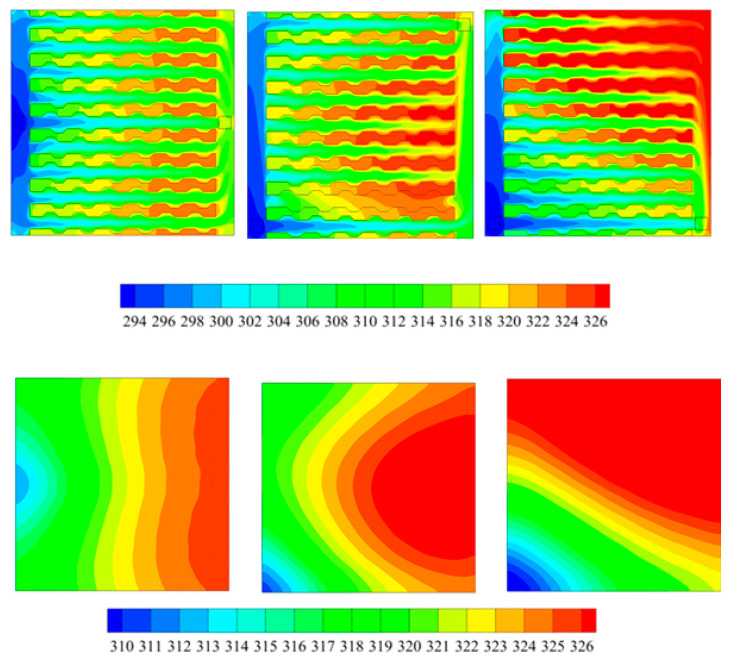
Temperature on the microchannel heat sink (MCSH) (**top**) and on the surface of solar cell (**bottom**); (left: I-type, middle: Z-type, right: C-type).

The layout of the MCHS will influence the thermal performance greatly [18,19]. Numerical simulations were carried out for the MCHS using 1 vol % Al_2_O_3_-water nanofluid (particle size 20 nm) when the flow rate at inlet being 0.225 cm^3^/s. The temperatures at the plane of Z = 0.25 mm and surface temperatures of solar cell were obtained (see in Figure 4). Figure 4 shows us that the I-type of MCSH demonstrated the most homogeneous temperature comparing with that in other type (Z-type and C-type). Therefore, the I-type of MCSH can be utilized to cool the surface of the solar cell.

### 3.2. Effect of Trapezoidal Groove

MCSH with trapezoidal grooves have better thermal performance than those with triangle grooves and rectangle grooves [21]. The structural parameters of trapezoidal groove were optimized. Nevertheless, only the temperature and Nusselt number of MCHS were analyzed in Ref. [34]. The interactions among temperature, particle concentration, flow, viscosity, thermal conductivity were not taken into account for analysis of the thermal performance of MCSH. In order to investigate the thermal performance of MCHS with a trapezoidal groove, one microchannel intercepted from the center of I-type MCHS was used as an object of study.

**Figure 5:**
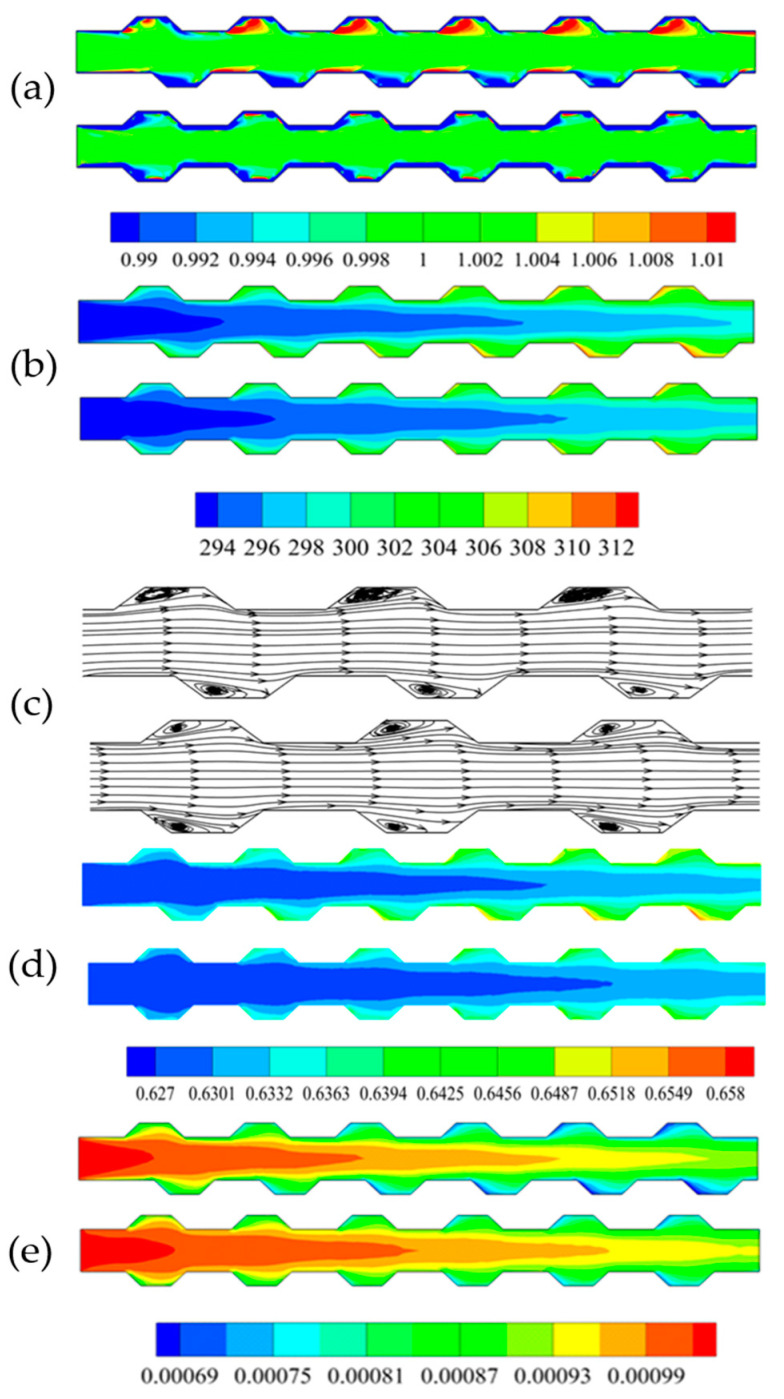
The particle concentration (**a**); temperature (**b**); streamlines (**c**); thermal conduction (**d**) and viscosity (**e**).

For the 1 vol % Al_2_O_3_-water nanofluid with a particle size of 20 nm, numerical simulations of MCHS with trapezoidal grooves of symmetric and staggered arrangements were carried out at a Reynolds number of 600. The calculated particle concentrations (non-dimensional volume fraction), temperatures, streamlines, viscosities, and thermal conductivities are shown in Figure 5a–e, respectively. It can be seen from Figure 5a that, there was a region possessing high particle concentration in every trapezoidal groove on the top side, this was because the particles in the vicinity of wall would be pushed away from the wall by thermophoretic force and Brownian force. Thereafter, these particles would stay at the interior of the vortex. This could be interpreted by combining Figure 5a,b for temperature distributions and Figure 5c for streamlines. Instead, a thinner high concentration region after every trapezoidal groove on the bottom side occurred, this was because all the particles pushed away from the wall in the region of groove by thermophoretic force would be taken away by the upstream flow when the vortex in groove region was moderate. Figure 5d shows us that the thermal performance of MCHS with symmetric arrangement trapezoidal grooves is better than that of MCHS with staggered arrangement trapezoidal grooves. This was basically consistent with what is shown in Figure 5b. Figure 5e shows us that the the viscosity in the middle region was higher than that in the region near the wall, because the temperature near the wall was much higher than that of the channel center. What makes us wonder is that a higher particle concentration region in the trapezoidal groove did not result in a higher viscosity region. This is because of a greater contribution of temperature than that of particle concentration, which could be interpreted by combining Equations (7) and (8).

**Figure 6:**
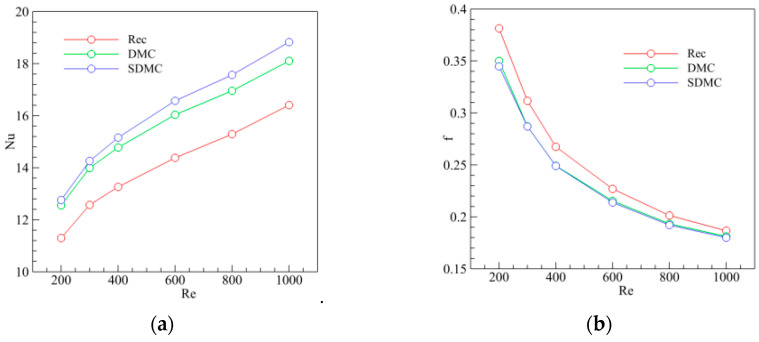
Effect of trapezoidal groove on the Nusselt number and friction coefficient. (**a**) Nusselt number vs. Reynolds number; (**b**) Friction coefficient vs. Reynolds number.

In order to compare the thermal performance of MCHS with and without a trapezoidal groove, the Nusselt number and friction coefficient of MCHS were calculated at Reynolds number 200, 300, 400, 600, 800, and 1000 (see in Figure 6a,b). Figure 6a shows us that the thermal performance of MCHS with a trapezoidal groove was better than that of MCHS with a rectangle channel (Rec). In addition, the thermal performance of MCHS with staggered arrangement of trapezoidal grooves (SDMC) was slightly better than that of MCHS with symmetric arrangement trapezoidal grooves (DMC). The likely reasons are that, (1) heat boundary layers were being destroyed periodically; (2) the vortex in the groove took the heat from the wall away to enhance heat transportation; (3) the increasing interfacial area between solid and fluid. However, the friction coefficient in MCHS with grooves is larger than that in rectangle channel without grooves (Figure 6b). The friction coefficients in MCHS with staggered arrangement grooves and that in MCHS with symmetric arrangement grooves are almost the same. This means, the thermal performance of MCHS with staggered arrangement grooves is better because of the higher Nusselt number.

**Figure 7:**
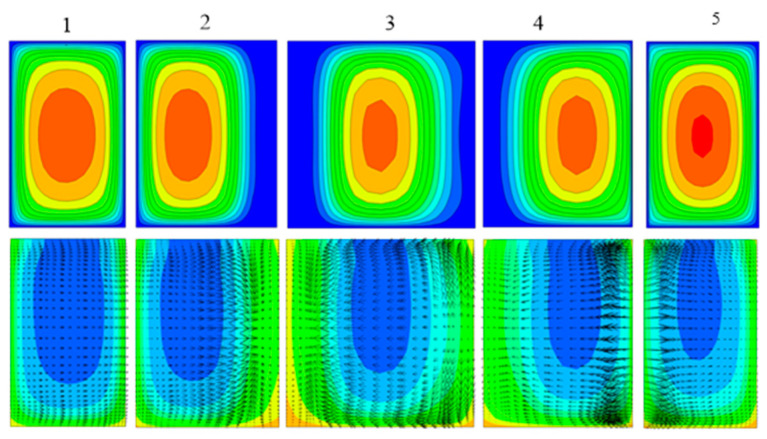
Effect of groove on the temperature and velocity (**top**) Velocity contour map; (**bottom**) Temperature and velocity vector.

To gain insight into the effect of trapezoidal grooves on the thermal performance, the velocity contour map, temperature distribution and velocity vector in five planes normal to the main flow(Y-Z plane) were analyzed after the numerical simulations of MCHS with 1 vol % nanofluid (particle size 20 nm) at Re = 600. Figure 7a shows us that the centers of flow pattern drifted from the center to left at first, and then drift to right, and finally back to the center. This shift of the centers of flow pattern can induce blinking flow to generate chaotic mixing for heat transfer enhancement [43]. Figure 7b show us that the area occupied by cool fluid in plane-5 is obviously smaller than that in plane-1, that is, more heat is pushed away from the wall to the channel center by the staggered arrangement of trapezoidal grooves, in other words enhancing the heat transfer. This can be attributed to the secondary flow at every plane normal to the main flow.

### 3.3. Discussion on the Relation of Brownian and Thermophoretic Diffusion

**Figure 8:**
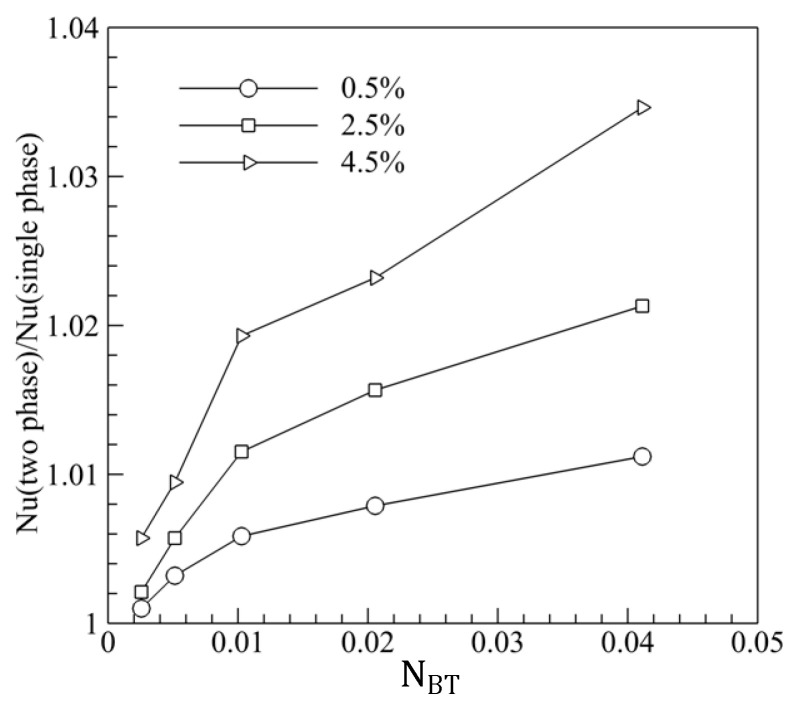
The effect of the movement of nanoparticle on the heat transfer.

It is known to all that Brownian motion and thermophoresis of nanoparticles in nanofluid are two most important mechanisms to enhance heat transfer. The effect mechanisms of Brownian motion and thermophoresis on the heat transfer are not the same. It is significant to analyze the effect of trapezoidal groove on the thermal performance of MCHS. The Nusselt numbers in MCHS with trapezoidal grooves were calculated for 0.5%, 2.5%, 4.5% vol nanofluid, the nanoparticles being 80 nm, 40 nm, 20 nm, 10 nm, and 5 nm (Figure 8). $N_{BT}$ represents the ratio of Brownian diffusion and thermophoretic diffusion, and can be written as $N_{BT}=D_{B}T\rho_{f}/\beta \mu_{f}\Delta T$ [31]. It can be seen from Figure 8 that, (1) The Nusselt number of two-phase model was always larger than that of single-phase model, this agrees with that in [44]. This is because Brownian motion and thermophoresis of the nanoparticles in nanofluid enhance the heat transportation; (2) The larger volume fraction of nanofluid could enhance heat transfer because more nanoparticles took part in heat transportation; (3) Larger $N_{BT}$ due to smaller particle size induced larger heat transfer enhancement because of more violent Brownian motion when the other conditions were fixed. However, there was a transition point at the particle size being 20 nm ($N_{BT}$ = 0.01). This reason for this was that more heat was transported by thermophoresis when the particle size was bigger than 20 nm, while more heat was transported by Brownian motion when particle size was smaller than 20 nm.

### 3.4. Impacting Factors on the Thermal Performance of MCHS

Numerical simulations of heat transfer in MCHS using 0.0%, 0.5%, 1.0%, 1.5%, and 2.5% vol Al_2_O_3_-water nanofluid at Re = 200, 300, 400, 600, 800, and 1000 were carried out, and the particle concentration, temperature distribution, and velocity vector map was obtained to analyze the mechanism of heat transfer enhancement in MCHS with trapezoidal grooves.

**Figure 9:**
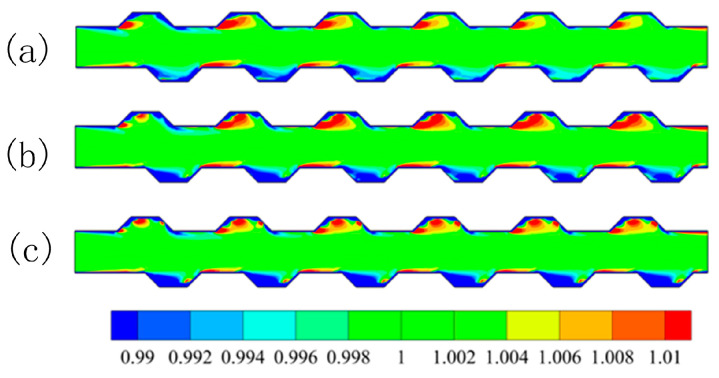
Non-dimensional concentration at various Reynolds numbers (Re). (**a**) Re = 200; (**b**) Re = 600; (**c**) Re = 1000.

**Figure 10:**
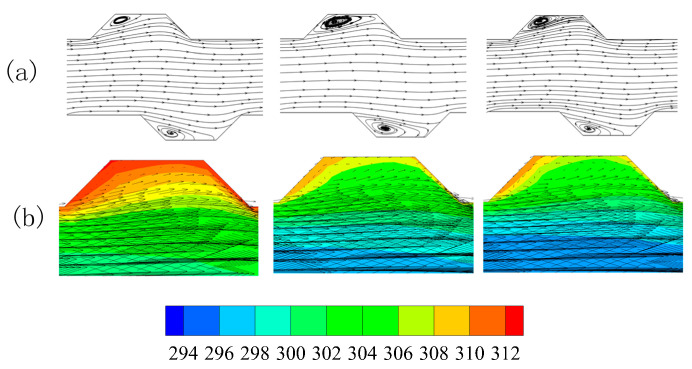
(**a**) Streamlines and (**b**) temperature in trapezoidal groove at various Re (Left: Re = 200; Middle: Re = 600; Right: Re = 1000).

The particle concentrations at Re = 200, 600, 1000 shown in Figure 9 tell us that, there was a high concentration region in every trapezoidal groove on the top side, there was instead a thinner high concentration region in the rear of groove. The reasons are discussed in Section 2.2. However, another focus is that, the morphology of the high region changed with the Reynolds number. The high concentration region was located in the front half of the groove (Figure 9a) at a lower Reynolds number (Re = 200). The high concentration region pervaded almost the entire groove at a high Reynolds number (Re = 1000), and was stretched into three sub-regions. (Figure 9c). Deduced by analogy, the high concentration region occupied the front and mid region of the groove at a moderate Reynolds number (Re = 600, Figure 9b). The temperature and velocity in the region of the groove should be presented in order to analyze the particle concentration specified above. Figure 10a shows us that for a smaller temperature gradient and vortex in the region of the groove at a lower Reynolds number, only a few particles were pushed by thermophoresis away from the wall, and fewer particles diffused and flowed with the upstream direction. On the contrary, greater temperature gradients and larger vortexes were present in the region of the groove, leading to more particles being pushed away from the wall, and being located stably in the entire groove. The particles in three sub-regions were driven by thermophoresis from the three edges of the trapezoidal groove.

**Figure 11:**
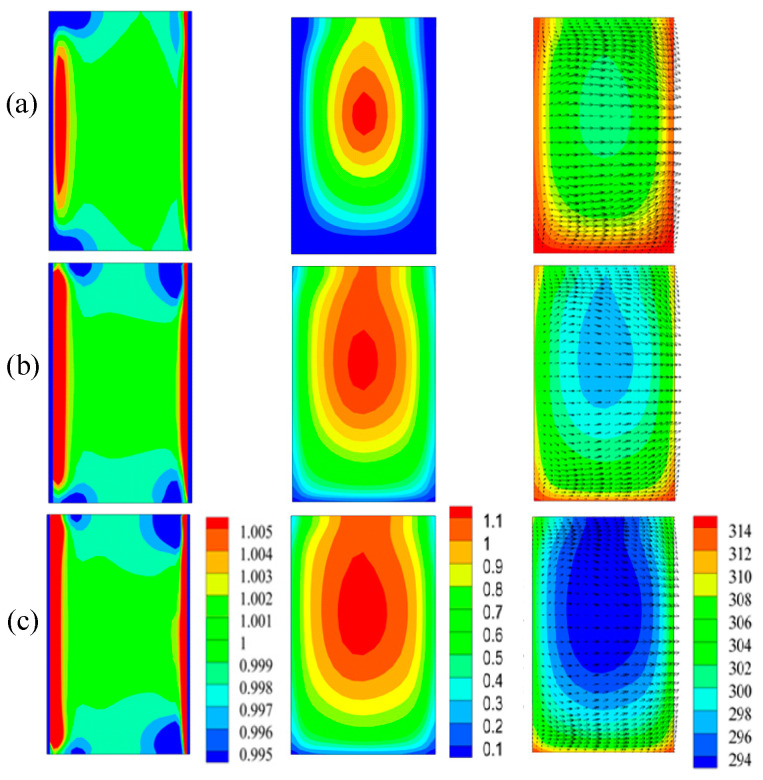
The particle concentration (left column), viscosity (middle column), temperature and velocity (right column) at various Reynolds number ((**a**) Re = 200; (**b**) Re = 600; (**c**) Re = 1000).

The particle concentration, temperature, viscosity, velocity in the planes normal to the main flow should be analyzed in order to understand the effect of the trapezoidal groove on the heat transfer, at various Reynolds numbers. It can be seen from Figure 11 (left) that the concentration uniformity of nanoparticles was better at lower Reynolds numbers than that at higher Reynolds numbers. The likely reason for this is that higher fluid temperatures result in higher thermal conductivity and lower viscosity, and nanoparticles homogenized according to Brownian diffusion, because Brownian diffusion prevails over thermophoretic diffusion according to the equation $N_{BT}\propto kT/\mu$ when $N_{BT}$ is greater. The single-phase model could be applied at a lower Reynolds number. It is known from Equation (8) that viscosity was related to temperature and particle concentration; however, it can be seen from Figure 11 (middle) that combining Figure 11 (right), the viscosity where temperature was higher, was instead lower. The likely reason for this is that the effect of the nanofluid temperature on the viscosity prevailed over that of particle concentration.

**Figure 12:**
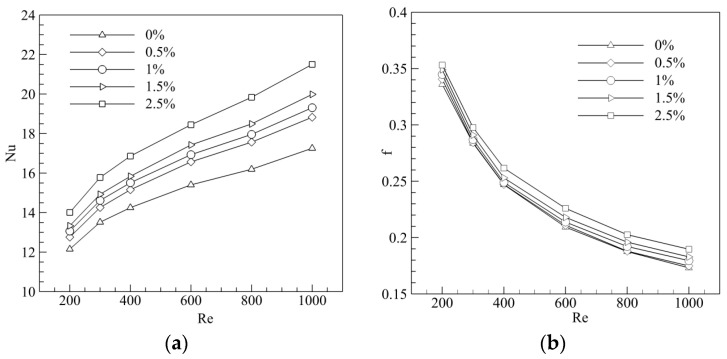
Effect of volume fraction on the (**a**) Nusselt number (Nu) and (**b**) base fluid (f).

The effect of added nanoparticles on the Nusselt number and friction coefficient are presented in Figure 12 for the 0.0%, 0.5%, 1.0%, 1.5%, 2.5% vol Al_2_O_3_-water nanofluid suspending 20 nm nanoparticles at various Reynolds numbers. It can be seen that the Nusselt number increased with the Reynolds number and volume fraction, because more heat could be transported by the movement of more nanoparticles (with the inclusion of Brownian motion and thermophoresis) and more convection of base fluid. Figure 12b shows us that the friction coefficient decreased with the increase of the volume fraction due to greater viscosity.

**Figure 13:**
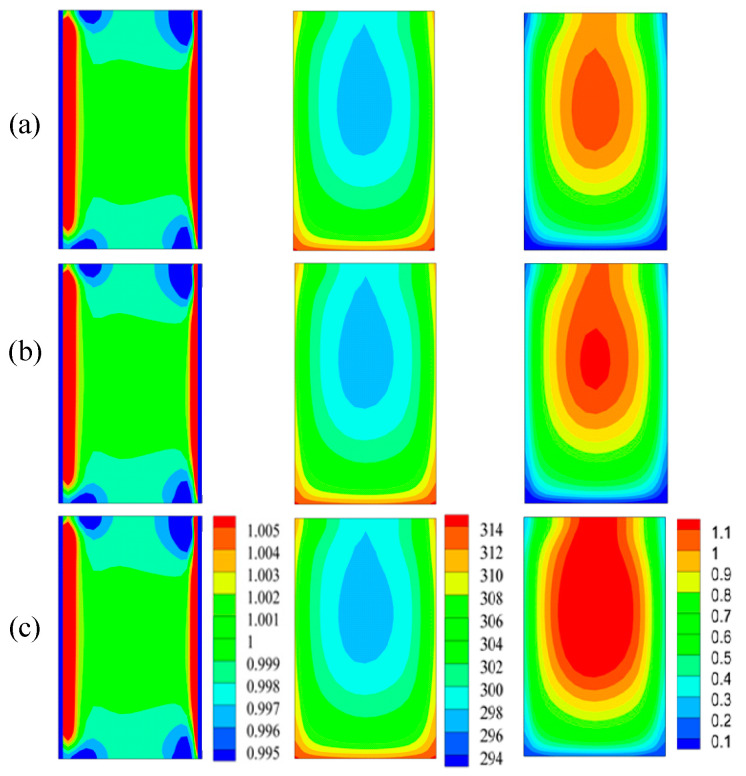
Particle concentration (left), temperature (middle), and viscosity (right) of nanofluids with various volume fractions (**a**) 0.5%; (**b**) 1.0%; (**c**) 2.5%.

Figure 13 shows us the non-dimensional particle concentration, temperature, and viscosity in the plane-6 (cf. Figure 2) used to study the effect of nanoparticles on the thermal conductivity. It can be seen from left column of Figure 13, the particle non-dimensional concentrations were almost same for three volume fractions. Nevertheless, better uniformity of temperatures (middle column) are presented, and this means better thermal conductivity. The right column of Figure 13 showed greater viscosity for 2.5% volume nanofluid. Combining Figure 13 (middle column), it can be concluded that the effect of volume fraction on viscosity dominated the factor of temperature.

**Figure 14:**
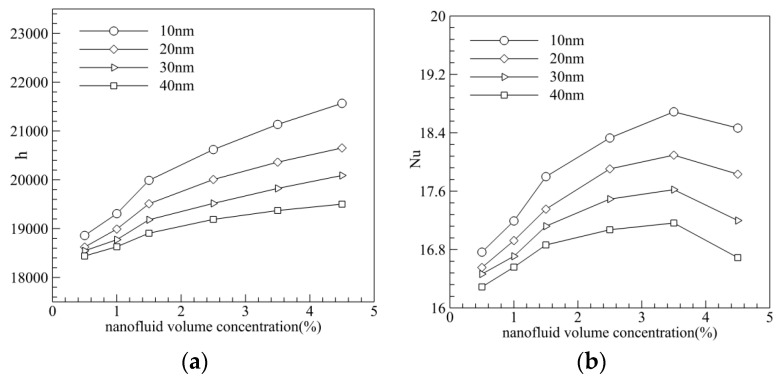
Effect of particle size on heat transfer. (**a**) Effect of particle size on heat transfer coefficient (h); (**b**) Effect of particle size on Nu.

It is known from the analysis in 2.3 that particle size affects heat transfer efficiency by means of Brownian motion and thermophoresis. The heat transfer coefficient and Nusselt number of 0.5%, 1.0%, 1.5%, 2.5%, 3.5%, and 4.5% vol nanofluid was calculated for Reynolds number being 600. It can be seen from Figure 14a that heat transfer coefficient increased with the volume fraction of the nanofluid, decreasing instead with the particle size. Figure 14b shows us also that smaller a particle size produced a greater Nusselt number. However, the Nusselt number increased with the volume fraction when the volume fraction was less than 3.5%, and decreased with the volume fraction when *φ* > 3.5%. The reason for this was likely due to a greater volume fraction resulting in greater effective thermal conductivity, but a smaller path for nanoparticles.

**Figure 15:**
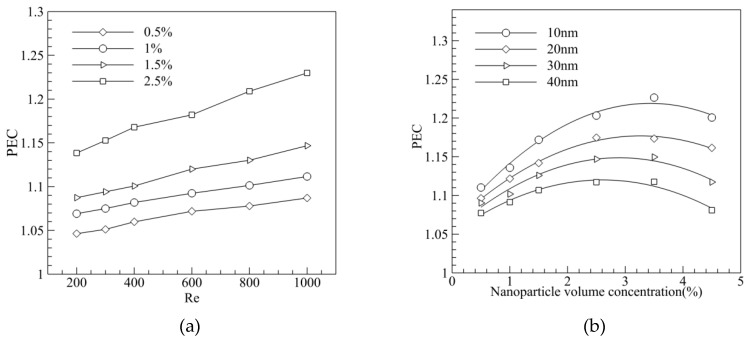
Effect of volume fraction and particle size on Performance Evaluation Criterion (PEC). (**a**) Effect of volume fraction on PEC; (**b**) Effect of particle size on PEC.

It is known from Figure 12 that, Nusselt number of MCHS increased with the Reynolds number, but the friction coefficient decreased with the Reynolds number. A comprehensive index of thermal performance of MCHS and PEC, considering the Nusselt number and friction coefficient, should be introduced. It can be seen from Figure 15a that the PEC (i.e., thermal performance) of MCHS increased with volume fraction and the Reynolds number. However, PEC dropped when the volume fraction exceeded 3–3.5% for various nanofluids with various particle sizes (Figure 15b), because the viscosity increased violently when the volume fraction was too great. In addition, the optimized volume fraction for smaller particles (10 nm) was greater (~3.5%), while the value was ~2.5% for bigger particles (40 nm).

### 3.5. Optimization of the Structural Parameters

**Figure 16:**
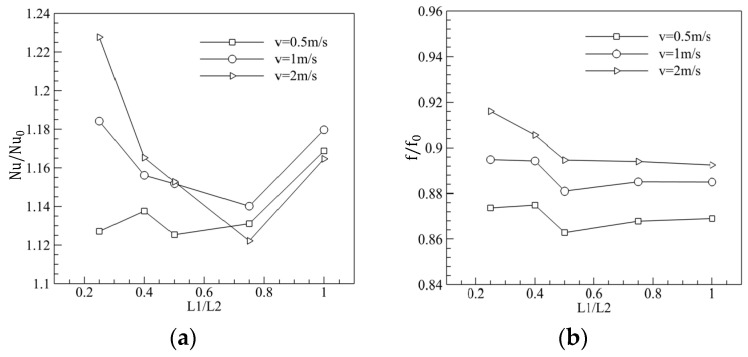
Effect of L1/L2 on the Nu and f (L3 = 0.75). (**a**) Effect on the Nusselt number; (**b**) Effect on the friction coefficient.

**Figure 17:**
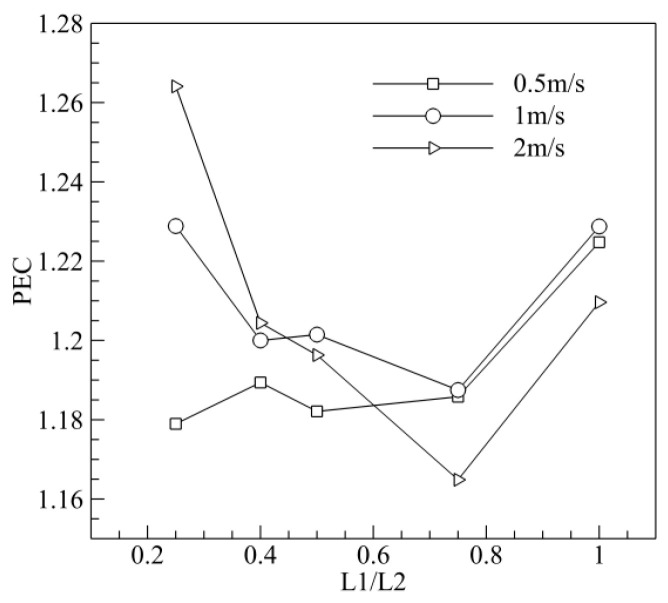
Effect of L1/L2 on the heat transfer (PEC) (L3 = 0.75).

To improve the thermal performance of MCHS with a trapezoidal groove, the structural parameters of the trapezoidal groove were optimized. Numerical simulations of MCHS with and without trapezoidal grooves were carried out for various geometrical parameters (L1/L2 = 0.25, 0.4, 0.5, 0.75, 1.0) and various inlet velocities (v_in_ = 0.5, 1.0, 2.0 m/s), and the Nusselt number, friction coefficient and PEC were calculated, where, the subscript was 0 for the rectangle channel without trapezoidal grooves. Figure 16a shows us that Nu/Nu_0_ was at the lowest point when L1/L2 ~ 0.75. Figure 16b shows that, f/f_0_ increased with L1/L2 when L1/L2 was less than 0.5, while f/f_0_ decreased with the increase of L1/L2 when L1/L2 was bigger than 0.5. Figure 17 shows us that PEC is at its lowest when L1/L2 was about 0.75, that is, the thermal performance of MCHS with trapezoidal grooves was worst at L1/L2 = 0.75.

## 4. Conclusions

Aiming at the MCHS used in micro solar cell, a numerical model for coupling calculation was established on the basis of the Buongiorno model after taking into account the interaction among particle concentrations, the temperature of the nanofluid, viscosity of the nanofluid, and the flow of the nanofluid. Numerical simulations of mass and heat transfer in MCHS with a trapezoidal groove for various volume fraction, and various particle sizes at various Reynolds number were carried out to obtain particle concentration, temperature, viscosity, and velocity of the nanofluid. The following conclusions can be drawn:

(1) The solar cell with I-type MCHS has the most homogeneous temperature distribution on the surface compared to that with Z-type or C-type MCHS.
(2) Higher heat transfer capability can be produced by a heterogeneous two-phase model than by a single-phase model. The thermal performance increases with N_BT_. The reason for this is that larger N_BT_ can induce much more microconvection to enhance heat transfer by Brownian motion when the particle size is smaller. The volume fraction has no relation to N_BT_.
(3) Thermal performance at an equal pump power as an index of heat transfer enhancement will increase with an increase in the Reynolds number and volume fraction. There is an optimum volume fraction to every particle size. A smaller optimum volume fraction corresponds to smaller particle size.
(4) The heat transfer performance is the worst when the parameter of the trapezoidal groove L1/L2 is 0.75, and it should be avoided in the MCHS design.

## Figures and Tables

**Table 1:** The parameters of materials.

	Thermal Conductivity (W/m·K)	Density (kg/m^3^)	Specific Heat (J/kg·K)
photovoltaic modules	59	5320	310
PCB plate	107	2610	904

**Table 2:** Material property of nanoparticle and base fluid.

	*ρ*/kg/m^3^	*k*/W/m·K	*c_p_*/J/kg·K
Water	988.2	0.6	4182
Al_2_O_3_	3970	42	880
